# Effect of Single Administration of Coffee on Pupil Size and Ocular Wavefront Aberration Measurements in Healthy Subjects

**DOI:** 10.1155/2016/9578308

**Published:** 2016-06-29

**Authors:** Handan Bardak, Murat Gunay, Ugur Mumcu, Yavuz Bardak

**Affiliations:** ^1^Department of Ophthalmology, Haydarpasa Numune Training and Research Hospital, Uskudar, 34668 Istanbul, Turkey; ^2^Department of Ophthalmology, Zeynep Kamil Maternity and Children's Disease Training and Research Hospital, Uskudar, 34668 Istanbul, Turkey

## Abstract

No study has so far evaluated the impact of coffee drinking on ocular wavefront aberration (OWA) measurements. This study presents novel findings regarding the OWA of the eye following coffee intake. We aimed to evaluate the acute changes in pupil size and OWA of the eye after single administration of coffee. A total of 30 otherwise healthy participants were included in this prospective study. All subjects drank a cup of coffee containing 57 mg caffeine. Measurements of pupil size, total coma (TC), total trefoil (TF), total spherical aberration (TSA), and total higher order aberration (HOA) were performed before and at 5 minutes, at 30 minutes, and at 4 hours after coffee drinking using a wavefront aberrometer device (Irx3, Imagine Eyes, Orsay, France). The mean age of the study population was 20.30 ± 2.74 years. Pupil size did not show a significant change during the measurements (*p* > 0.05). A significant increase was observed in TF and HOA measurements following coffee intake (*p* = 0.029 and *p* = 0.009, resp.). Single administration of coffee results in significant increase in TF and total HOAs in healthy subjects without any effect on pupil diameter. Ultrastructural changes in the cornea following coffee intake might be of relevance to the alterations in ocular aberrations in healthy subjects.

## 1. Introduction 

Caffeine is a methylxanthine derivate alkaloid found in many food and various beverages such as coffee, tea, and cola-flavoured drinks. Caffeine has been demonstrated to be associated with increased blood pressure, decreased heart rate, and reduced cerebral blood flow. The level of caffeine content was reported to be highest in coffee among the dietary products [1, 2]. Also caffeine has been shown to be associated with changes in intraocular pressure (IOP), retinal and choroidal thickness, and pupil size [3–6].

The dual innervation of the iris has already been demonstrated. It has been known that constriction of the pupil is related to parasympathetic outflow from the Edinger-Westphal nucleus. Studies have also shown cholinergic stimulation of the constrictor muscle and alpha-adrenergic stimulation of the dilator muscle [7, 8].

The quality of the retinal image in focus depends on the optical quality of the eye. Ocular wavefront aberrations (OWA) have been reported to have an impact on retinal image quality of the human eye [9, 10]. Lower order WA including spherocylindrical refractive errors could be measured with conventional phoropters and autorefractors and can easily be corrected with spectacles or contact lenses. However, higher order WA cannot be corrected with conventional methods and are decomposed and calculated from Zernike polynomials [11, 12]. The relationship between pupil size and OWA has been suggested previously. It has been stated that optical quality is less influenced by optical aberrations with smaller pupil sizes [13].

The Irx3 wavefront aberrometer device measures the entire optical system of the eye in a fast one-shot operation and displays advanced data formats including wavefront maps and Zernike coefficients. The measurements obtained from this device have been reported to be repeatable and comparable with other aberrometers used in the current clinical practice [14].

To the best of our knowledge, no study exists regarding the effect of caffeine on OWA measurements. The purpose of this study is to evaluate the acute effect of coffee on OWA along with pupil size measurements in healthy subjects.

## 2. Materials and Methods

A total of 30 young, healthy, and nonregular coffee drinker volunteers from the staff of Haydarpasa Training and Research Hospital were recruited for the study. A local ethical approval was obtained from the institutional review board. The study followed the principles laid out in the Declaration of Helsinki. Participants were not regular and chronic coffee drinkers. None of the participants had any history of smoking, drug or alcohol use, ocular and/or systemic disease, previous ocular surgery, and/or trauma.

The participants were instructed not to take any medications and consume caffeine containing drinks for at least 12 hours. A complete baseline ophthalmic examination was performed in all subjects including best corrected visual acuity (BCVA) assessment, refractive measurements, slit lamp biomicroscopy, IOP measurement, and fundus evaluation. Subjects with spherical refractive power between −1.0 D and +1.0 D and cylindrical refractive power under 1.0 D were included for the study. Subjects were then invited for OWA measurements.

Participants received one cup of Turkish coffee which was prepared in a way as indicated in the previous literature [5]. Pupil size and OWA measurements were assessed before coffee drinking and at 5 minutes, at 30 minutes, and at 4 hours after coffee drinking by using a wavefront aberrometer device (Irx3, Imagine Eyes, Orsay, France).

### 2.1. Measurements of Ocular Wavefront Aberrations

An experienced physician performed all the measurements. The physician paid attention to limit head movements of the subjects to a minimum during the procedures. During the measurements the pupil diameter was automatically recorded by the device. All measurements were carried out under the same room illumination. All measurements were performed under natural pupil size.

The Irx3 wavefront aberrometer follows the Hartmann-Shack principles to measure the whole suite of ocular aberrations. While the subject fixates on a target inside the device, a fine beam of infrared light with a wavelength of 780 nm is directed towards the fundus. The beam is backscattered by the retina and travels back through the eye and pupil. The exit beam is then directed to a Hartmann-Shack wavefront sensor that analyses the shape of the optical wave.

The Irx3 machine creates an OWA map and Zernike graph. Zernike polynomial series up to and including the 10th order was calculated for each measurement. The root-mean-square (RMS) wavefront error of each Zernike mode was detected and the amounts of total coma (TC), total trefoil (TF), total spherical aberration (TSA), and total higher order aberrations (HOAs) were determined.

### 2.2. Statistical Analysis

The NCSS (Number Cruncher Statistical System, 2007, Kaysville, Utah, USA) programme was used for the statistical analysis. Descriptive statistics were given as mean and standard deviation. Friedman test or ANOVA and repeated measurements tests with Wilcoxon-Signed Ranks and Bonferroni tests as pairwise comparisons were used depending upon the distribution of the data. Significance was assessed at the levels of *p* < 0.05.

## 3. Results 

There were 14 male (46.7%) and 16 female (53.3%) participants in the study. The mean age of the study population was 20.30 ± 2.74 years (range, 18–26 years).

Although pupil size showed a slight increase following coffee drinking, it did not significantly change during the measurements (*p* > 0.05). Pupil size measurements are given in Table 1.

Regarding the OWA measurements, significant changes were present in TF and total HOA measurements following coffee intake (*p* = 0.029 and *p* = 0.009, resp.). Pairwise comparisons revealed that TF measurements were significantly higher at 5 minutes (*p* = 0.019) and 4 hours (0.002) after coffee as compared to baseline measurements. Also TF measurements at 4 hours were significantly higher compared to measurements at 30 minutes following coffee intake (*p* = 0.02). Furthermore, total HOA measurements at 5 minutes (*p* = 0.003) and 4 hours (*p* = 0.001) after coffee were significantly higher than the baseline total HOA measurements.

The TC (*p* = 0.409) and TSA (*p* = 0.09) measurements did not exhibit significant alterations. The OWA measurements are summarized in Table 2.

Correlation analysis was performed to identify any possible relationship between pupil size and OWA measurements at each time point. It was found that change in pupil size was significantly correlated with the changes in TF and total HOA at 5 and 30 minutes following coffee intake (*p* < 0.01). Furthermore, pupil size change was significantly correlated with the total HOA change at 4 hours after coffee drinking (*p* < 0.01). Correlation results are provided in Table 3.

## 4. Discussion

No study has so far studied the effect of caffeine on OWA measurements previously. In the present study, we did not observe any significant change in pupil size measurements following coffee intake. However, a cup of coffee resulted in increased TF and total HOAs. This result might indicate that single administration of coffee may be associated with decreased image quality by the effect on TF and HOA. We also found a positive correlation between the changes in pupil size and HOA measurements at each time point.

Caffeine stimulates central nervous system and cardiovascular system. It promotes myocardial stimulation, vascular resistance through vasoconstriction, blood pressure, bronchodilation, and basal metabolic rate [15, 16]. Caffeine also decreases cerebral and retinal blood flow with a significant increase in the resistivity index of the ophthalmic, central, and short posterior ciliary arteries in healthy volunteers [17, 18].

As indicated previously, caffeine promotes sympathetic noradrenaline discharge, thereby causing pupil dilatation but only in nonconsumers [19]. All participants in our study were not chronic coffee consumers. Although pupil size showed a slight increase following coffee administration, this finding did not reach a statistically significant level. Younger age, steady metabolic state, and adequate systemic regulatory mechanisms with relatively lower amount of coffee administration (57 mg) might contribute to insignificant changes in pupil size in our study.

Animal studies have found that caffeine, when applied topically, prevents apoptosis and removes reactive oxygen species in lens, thus having anticataractogenic effect [20, 21]. Moreover, it has been demonstrated that administration of caffeine causes changes of collagen fibers of Bowman's membrane and corneal stroma along with alterations in the amount of collagen fibers and stromal cells in developing chicken corneas [22]. Evereklioglu et al. [23] have shown that high dose of caffeine administration to pregnant rats leads to histopathological changes in the cornea of newborn rats including vacuolated endothelial cells with proliferation, hyperchromasia, polymorphism, endothelial cell agenesis, increased stromal mitotic activity, and focal increase in corneal thickness with widely separated corneal lamellae. No study exists in the literature regarding the impact of systemic caffeine administration on corneal structure. It has already been stated that corneal structural changes may result in biomechanical and aberrometric alterations in the eye [24]. It should also be thought that such changes in corneal shape mainly occur in the presence of a disrupted layout of the corneal collagen fibers [25]. We generated a new result in the present study that single administration of coffee induced significant increases in TF and total HOA in the eye of healthy individual. We may think that such effect might be related to the possible influence of caffeine on corneal shape which is associated with the compromise of the corneal collagen fiber array. However, strictly speaking, we could only identify significant changes in TF and total HOA without any change in TC and TSA. It would seem that the effect of caffeine on OWA should be evaluated in taking care with other corneal biomechanical properties such as corneal hysteresis and corneal resistance factor in order to better clarify the relationship between caffeine and OWA. Furthermore, accommodation status of the eye has been known to be related to WA [26]. One drawback of our study is that the accommodation of the patients was not assessed.

The WA have been reported to be different under different pupillary conditions [27]. Fan et al. [28] found that all the HOAs decreased statistically and significantly as the pupil size gets smaller. Supportively, in our study, we observed that changes in total HOAs were positively correlated with the changes in pupil size at each time point. The changes in total HOAs showed significant increase as the change in pupil size increases.

Regarding the pharmacokinetics of caffeine, Mort and Kruse [29] have stated that caffeine reaches its maximum plasma concentration in 20 minutes to 120 minutes with a systemic half-life of 3 to 6 hours. Vural et al. [5] studied the changes in choroidal thickness after administration of a cup of coffee and found that coffee causes a significant decrease in choroidal thickness, starting at 5 minutes after coffee drinking, and tended to persist for at least 4 hours. In the present study, alterations in TF and total HOAs are maintained for 4 hours following coffee intake.

We may propose that a cup of coffee may have an influence on visual quality by exhibiting alterations in TF and HOAs in the eye. Furthermore, it should be kept in mind that coffee may impact vision by its stimulating effect on central nervous system. Further prospective studies should be performed considering the possible effect of coffee on subjective and objective visual acuity.

## 5. Conclusions

Until recently no study has evaluated the effect of coffee intake on OWA measurements of the eye in healthy subjects. The present study showed that single administration of coffee results in significant increases in TF and total HOA measurements of the eye without any significant change in pupil diameter in healthy individuals. Changes in TF and total HOAs following coffee drinking might have an association with the changes in corneal ultrastructure. We suggested that further studies are necessitated to better clarify the underlying mechanism of the impact of caffeine on OWA measurements.

## Figures and Tables

**Table 1:** Pupil size measurements before and after coffee intake.

	Before	After 5 minutes	After 30 minutes	After 4 hours	^a^ *p*
Pupil size, mm	6.79 ± 0.10	6.98 ± 0.80	6.97 ± 0.66	6.77 ± 1.49	0.270

^a^ANOVA test with repeated measurements.

Results are denoted as mean ± standard deviation.

**Table 2:** RMS of the total ocular wavefront aberration measurements before and after coffee intake.

WA	Before	After 5 minutes	After 30 minutes	After 4 hours	^a^ *p*
TC	−0.10 ± 0.05	−0.12 ± 0.07	−0.12 ± 0.07	−0.13 ± 0.12	0.409
TF	−0.11 ± 0.06	−0.14 ± 0.07	−0.11 ± 0.07	−0.16 ± 0.12	*0.029*
TSA	−0.07 ± 0.10	−0.06 ± 0.10	−0.09 ± 0.11	−0.07 ± 0.17	0.090
HOA	0.57 ± 0.42	0.69 ± 0.61	0.66 ± 0.59	0.72 ± 0.60	*0.009*

^a^Friedman test, *p* < 0.05.

RMS: root-mean-square; WA: wavefront aberrations; TC: total coma; TF: total trefoil; TSA: total spherical aberration; HOA: total higher order aberration.

Results are denoted as mean ± standard deviation.

**Table 3:** Correlation results between the changes in pupil size and ocular aberration measurements.

	TC	TF	TSA	HOA
Pupil size 5 minutes (*r*; *p*)	−0.283; 0.131	−0.464; 0.10	−0.210; 0.911	0.787; <0.001^*∗*^
Pupil size 30 minutes (*r*; *p*)	−0.351; 0.057	−0.536; 0.002^*∗*^	−0.132; 0.488	0.591; 0.001^*∗*^
Pupil size 4 hours (*r*; *p*)	−0.054; 0.778	0.050; 0.793	−0.333; 0.072	0.376; 0.041^*∗*^

*r*: Spearman correlation coefficient, *p* < 0.05^*∗*^.

TC: total coma; TF: total trefoil; TSA: total spherical aberration; HOA: total higher order aberration.

Results are denoted as *r*; *p*.
